# Processing of stalled replication forks in *Bacillus subtilis*

**DOI:** 10.1093/femsre/fuad065

**Published:** 2023-12-05

**Authors:** Begoña Carrasco, Rubén Torres, María Moreno-del Álamo, Cristina Ramos, Silvia Ayora, Juan C Alonso

**Affiliations:** Department of Microbial Biotechnology, Centro Nacional de Biotecnología, CNB-CSIC, 3 Darwin Str, 28049 Madrid, Spain; Department of Microbial Biotechnology, Centro Nacional de Biotecnología, CNB-CSIC, 3 Darwin Str, 28049 Madrid, Spain; Department of Microbial Biotechnology, Centro Nacional de Biotecnología, CNB-CSIC, 3 Darwin Str, 28049 Madrid, Spain; Department of Microbial Biotechnology, Centro Nacional de Biotecnología, CNB-CSIC, 3 Darwin Str, 28049 Madrid, Spain; Department of Microbial Biotechnology, Centro Nacional de Biotecnología, CNB-CSIC, 3 Darwin Str, 28049 Madrid, Spain; Department of Microbial Biotechnology, Centro Nacional de Biotecnología, CNB-CSIC, 3 Darwin Str, 28049 Madrid, Spain

**Keywords:** replisome disassembly, RecA hub, SsbA hub, RNA polymerase hub, DNA damage tolerance, fork reversal, template switching

## Abstract

Accurate DNA replication and transcription elongation are crucial for preventing the accumulation of unreplicated DNA and genomic instability. Cells have evolved multiple mechanisms to deal with impaired replication fork progression, challenged by both intrinsic and extrinsic impediments. The bacterium *Bacillus subtilis*, which adopts multiple forms of differentiation and development, serves as an excellent model system for studying the pathways required to cope with replication stress to preserve genomic stability. This review focuses on the genetics, single molecule choreography, and biochemical properties of the proteins that act to circumvent the replicative arrest allowing the resumption of DNA synthesis. The RecA recombinase, its mediators (RecO, RecR, and RadA/Sms) and modulators (RecF, RecX, RarA, RecU, RecD2, and PcrA), repair licensing (DisA), fork remodelers (RuvAB, RecG, RecD2, RadA/Sms, and PriA), Holliday junction resolvase (RecU), nucleases (RnhC and DinG), and translesion synthesis DNA polymerases (PolY1 and PolY2) are key functions required to overcome a replication stress, provided that the fork does not collapse.

## Teaser

Different stresses interfering with DNA replication, collectively termed replication stress, pose a threat to genomic stability in *Bacillus subtilis* cells.

Abbreviations CD: Codirectional
c-di-AMP: Cyclic 3′, 5′-diadenosine monophosphate
DSBs: Double-strand breaks
dsDNA: Double-stranded DNA
DNAP: DNA polymerase
DDT: DNA damage tolerance
HJ: Holliday junction
HO: Head-on
RNAP: RNA polymerase
RTCs: Replication–transcription conflicts
ssDNA: Single-stranded DNA
TLS: Translesion synthesis
wt: Wild-type

## Introduction

In all living cells, replication fork progression can be compromised by both endogenous and environmental factors resulting in replication stress, which poses a threat to genomic stability (Cox et al. 2000, Ciccia and Elledge 2010, Zeman and Cimprich 2014). When the replisome encounters DNA lesions, secondary structures in the DNA template, tightly bound proteins, or when it clashes with RNA polymerase (RNAP) elongation complexes, transient fork stalling inevitably occurs. These stalled forks should be rescued for preventing fork degradation, allowing the resumption of DNA synthesis without chromosome breakage, and thus preserving genome integrity. Cells have evolved multiple mechanisms to maintain fork stability. However, how cells choose among these mechanisms remains to be elucidated. Here, we provide an overview of the mechanisms used by *Bacillus subtilis*^1^ cells to cope with replication stress.

During the replication of the circular genome in *B. subtilis* cells, DNA is synthesized by an essential multiprotein complex known as the replisome. DNA replication initiates with strand separation at the replication origin (*oriC*), where a pair of replisomes, which travel in opposite directions, are assembled. Replication ends at the terminus region (*terC*), when the two convergent replisomes meet and clash with the polar replication fork trap system (i.e. the RTP protein bound to the *terC* region), causing replisome dissociation (reviewed in Murray et al. 2017). During rapid growth, bacterial DNA replication occurs in an overlapping manner, termed multifork replication, wherein several rounds of replication begin before the first-round is completed. The *B. subtilis* replisome is organized into three functional groups: (i) the PolC holoenzyme, which is the replicative DNA polymerase (DNAP), is composed of several subunits (see below); (ii) the primosome complex, which comprises the replicative DNA helicase DnaC (functional homolog of DnaB*_Eco_*), the DNA primase DnaG, and the error-prone translesion synthesis (TLS) DNAP DnaE (absent in *Escherichia coli*) (Bruck et al. 2003); and (iii) the single-stranded binding protein SsbA (functional homolog of SSB*_Eco_*) that rapidly coats the single-stranded (ss) DNA and spreads over the consecutively exposed Okazaki fragments (Bruck et al. 2003, Sanders et al. 2010, Murray et al. 2017, Seco and Ayora 2017). The bulk of DNA synthesis is carried out by the PolC holoenzyme. This enzyme is organized into three discrete essential components: (i) PolC, which is composed of two domains (5′ → 3′ polymerase and proofreading 3′ → 5′ exonuclease); (ii) the processivity sliding clamp, DnaN (also known as β-sliding clamp), and (iii) the clamp loader complex (also known as  τ-complex) comprising the DnaX, HolA, and HolB subunits (Sanders et al. 2010, McHenry 2011, Seco et al. 2013, Murray et al. 2017, Seco and Ayora 2017).

The fundamental aspects of DNA replication are remarkably conserved (O’Donnell et al. 2013), but the replicative DNAP of bacteria of the Firmicutes Phylum (i.e. the PolC holoenzyme) cannot initiate DNA synthesis from an RNA primer (Sanders et al. 2010, Seco and Ayora 2017). PolC relies on DnaG and DnaE to initiate both leading- and lagging-strands synthesis (Seco and Ayora 2017). In an *in vitro* reconstituted replication system, DnaG synthesizes *de novo* a short RNA primer, which is briefly extended by DnaE before handing this chimeric RNA–DNA hybrid primer to the PolC enzyme (Sanders et al. 2010, Seco et al. 2013, Seco and Ayora 2017). The PolC holoenzyme replicates the genome with high accuracy, suggesting that it proofreads any mis-incorporated nucleotides and catalyzes synthesis of both leading- and lagging-strands (Sanders et al. 2010, Paschalis et al. 2017, Seco and Ayora 2017). Similarly, mammalian cells initiate DNA synthesis of both strands from hybrid RNA–DNA primers, which are synthesized by the Polα-primase complex (Pellegrini 2012).

**Figure 1.**
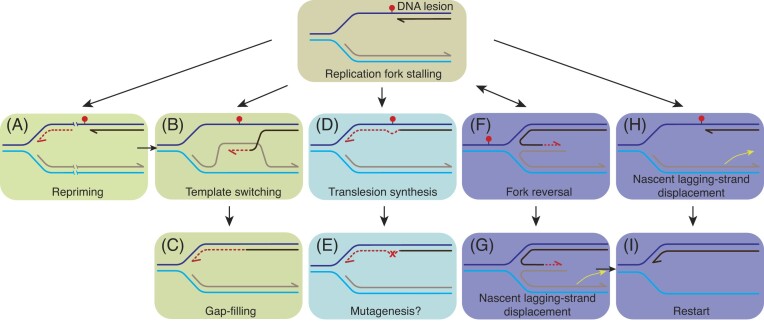
Potential replication stress response mechanisms. A replicative DNAP cannot accommodate a damaged template base (represented by a red dot) and transiently stalls. Replication may proceed *via* error-free (template switching, fork remodeling) or error-prone DDT pathways to allow replication to resume (A)–(E) or restart (F)–(I). The replicative DNAP may skip the lesion, and upon reloading of the primosomal complex and repriming, DNA synthesis continues. The resulting lesion-containing gap left behind is filled by template switching, mainly *via* a RecA-dependent mechanism (A)–(C). The replicative DNAP may be replaced by a specialized TLS DNAP that often incorporates an erroneous nucleotide opposite the damaged template, leading to mutagenesis (denoted by x) (D) and (E). Enzyme-catalyzed reversal of the stalled fork by annealing the nascent strands occurs, with the nascent leading-strand extended (F). The fork can be restored by regressing the reversed fork, or the nascent lagging-strand is removed to generate a 3′-fork DNA for replication restart (G)–(I). Alternatively, the nascent lagging-strand is removed to generate a 3′-fork DNA for replication restart (H) and (I).

Recent results suggest that in *B. subtilis* cells, the replisome often stalls and disengages from the replication fork in response to replicative stress (Fig. 1) (Mangiameli et al. 2017). In vertebrates and in some bacteria, upon replication stress, the PrimPol enzyme synthesizes a DNA primer downstream of lesions or at stalling structures to allow replication restart (Mouron et al. 2013, Bainbridge et al. 2021), but *B. subtilis* lacks a PrimPol-like repriming mechanism. To deal with replicative stress, *B. subtilis* may promote the removal or bypass of the barriers, and resume DNA replication at stalled forks by DnaG:DnaE-mediated repriming (see Fig. 1A–E), or may remodel the stalled fork (Fig. 1F–H) (Stoy et al. 2023). In the fork reversal process (also termed fork regression), the stalled fork is pushed backward, resulting in nascent complementary strands annealing to generate a protective four-way junction resembling a Holliday junction (HJ) structure (Fig. 1F) (Atkinson and McGlynn 2009, Neelsen and Lopes 2015, Bianco and Lu 2021). In a reversed fork, the lesion is placed on duplex DNA, facilitating its repair by specialized pathways before fork restoration. Fork remodeling proteins generate specific DNA branched structures for replication restart. Alternatively, fork reversal does not occur, and there is a displacement of the nascent lagging-strand to generate a ssDNA region where the helicase DnaC can be loaded (Fig. 1H). Mammalian cells frequently employ replication fork reversal to rescue a replication stress (Neelsen and Lopes 2015, Berti et al. 2020). In both *B. subtilis* and mammalian cells, the recombinase (RecA or Rad51, respectively) is consistently present at the stalled replication fork (Simmons et al. 2007, Lenhart et al. 2014, Zellweger et al. 2015).

To load the replicative helicase DnaC at this region of the chromosome outside *oriC*, four essential functions are required: three preprimosomal (PriA, DnaD, and DnaB) proteins and a chaperone-specific (DnaI) protein. First, PriA recognizes and binds to these branched structures and interacts with and loads DnaD and DnaB. These preprimosomal proteins in concert with DnaI load DnaC onto the template lagging-strand (Marsin et al. 2001, Polard et al. 2002, Velten et al. 2003, Bruand et al. 2005, Smits et al. 2011). Finally, DnaC and SsbA, acting as protein-interaction hubs, recruit the remaining proteins for replisome reassembly (Sanders et al. 2010, Seco et al. 2013).

In the evolutionarily distant bacterium *E. coli*, damage-induced fork stalling often results in the uncoupling of the replicative DNAP (PolIII holoenzyme) from the replicative DNA helicase (DnaB) (Cox et al. 2023). Subsequently, DnaB*_Eco_*, which is stably bound to the template lagging-strand, continues unwinding in a 5′ → 3′ direction, albeit much more slowly, and this generates a ssDNA region (O’Donnell 2006, Lewis et al. 2017). Finally, PolIII*_Eco_* skips the barrier and resumes DNA synthesis, leaving a lesion-containing gap behind (Fig. 1A) (reviewed in Cox et al. 2023). In wild-type (wt) *E. coli* cells, fork remodeling seems to be mainly triggered by head-on (HO) collisions at replication–transcription conflicts (RTCs) (reviewed in Marians 2018, Cox et al. 2023). Thus, the nature of the roadblock can, at least in part, dictate the fate of the stalled fork.

This review focuses primarily on the *B. subtilis* homologous recombination proteins required to cope with replication stress at stalled forks. We have summarized the genetic, cytological, and biochemical findings regarding these *B. subtilis* proteins. Other homologous recombination proteins involved in processing double-stranded DNA breaks (DSBs), which are formed when the replication fork encounters a nick in the template DNA, are beyond the scope of this manuscript. We direct readers to recent in-depth reviews for a comprehensive understanding of the repair mechanisms operating at DSBs (Ayora et al. 2011, Kowalczykowski 2015, Michel et al. 2018).

## Recombination proteins necessary to survive a replication stress in haploid spores

Over the last decades, numerous repair-by-recombination functions required to overcome replication stress have been identified and their roles in repair when multiple chromosomes and replication forks are present have been proposed. The multiple forms of differentiation and development of *B. subtilis* cells (e.g. sporulation and/or its revival) can be used to simplify the outcome and analyze DNA damage and replication stress when only one chromosome is present.

Spores are dormant cells containing only one inert chromosome. In response to nutrients and certain non-nutritional agents, a dormant mature haploid spore is synchronously resurrected (reviewed in Stragier and Losick 1996, Errington 2003, Setlow and Christie 2023). Upon adding spores to the germination medium, which marks time zero, spores transition through discrete and ordered timeline steps for returning to vegetative growth: germination (0–10 min), ripening (10–70 min), early (70–90 min) and late outgrowth (90–150 min), and burst (beyond 150 min) (Keijser et al. 2007, Sinai et al. 2015, Swarge et al. 2020, Zhou et al. 2022, Setlow and Christie 2023). DNA lesions that have accumulated in dormant spores must be repaired by specialized functions in the ripening period (Setlow and Christie 2023).

To gain new insights into the functions necessary to cope with replication stress when there is only one copy of the chromosome, the DNA of mature haploid spores, defective in one or more repair-by-recombination function(s), is damaged by ionizing radiation (IR). Subsequently, the predamaged inert haploid spores are revived under unperturbed conditions. IR treatment leads to several DNA lesions in a dose-dependent manner, including damaged template bases, single strand breaks and two-ended DSBs (Setlow and Christie 2023). During the ripening stage, the IR-induced damaged template bases on the unreplicated haploid genome are removed by base excision repair, the single nicks are repaired by LigD-dependent or LigD-independent pathways, and the two-ended DSBs are simply reconnected *via* Ku (also termed YkoV)- and LigD-dependent nonhomologous end joining (Weller et al. 2002, Wang et al. 2006, de Ory et al. 2016, Setlow and Christie 2023). Potentially antagonistic recombinational proteins do not compete these repair processes, because proteomic and transcriptomic analyses have shown that the helicases and nucleases involved in end resection steps in vegetative growth (e.g. AddAB, RecJ, and RecQ or RecS) are synthesized at later stages of spore revival (Keijser et al. 2007, Nicolas et al. 2012, Sinai et al. 2015, Swarge et al. 2020). During the early outgrowth stage, the synthesis of all DNA replication and of many recombination proteins takes place, and DNA replication initiates at *oriC* (*∼*90 min) (Sinai et al. 2015, Swarge et al. 2020). Remarkably, several proteins involved in the repair of RTCs are found to be overexpressed during the early outgrowth stage, including RecA, PcrA, Rnase J1, RnhC, and DNA topoisomerases (Table 1) (Keijser et al. 2007, Nicolas et al. 2012, Sinai et al. 2015, Swarge et al. 2020), with the latter enzymes providing a favorable DNA topology for replication initiation.

**Table 1.** Genes required to survive replication stress in *B. subtilis* cells and their *bona fide* counterparts in *E. coli*.

*B. subtilis* ^a^	Alternative name^b^	*E. coli* ^c^	Role of gene product
*recA*	*recE*4, *recB*3, *recP*149	*recA*	Strand exchange, ATPase, interacting hub
*ssbA* ^d^	–	*ssb*	Mediator, ssDNA binding, interacting hub
*recO*	*recL*16	*recO*	Mediator, binds and anneals ssDNA
*recR*	*recM*13, *recD*43	*recR*	Mediator, binds ssDNA and dsDNA
*radA* ^e^	*sms*	*radA* ^e^	Mediator, HJ binding, 5′→3′ helicase
*recF*	*rec*15	*recF*	Modulator, binds ssDNA–dsDNA
*rarA*	*yrvN*	*rarA*	Modulator, ATPase
*recX*	*recH*342	*recX*	Modulator
*recD*2	*yrrC*	No	Modulator, 5′→3′ DNA helicase
*pcrA* ^d,f^	–	*uvrD*	Modulator, 3′→5′ DNA helicase, backtracks RNAP
*ruvAB*	*recB2–ruvB*	*ruvAB*	Branch migration translocase
*recU* ^g^	*recG*40, *recV*40	*ruvC*	HJ resolvase, modulator
*recG*	*ylpB*	*recG*	Branch migration translocase
*lexA*	*dinR*	*lexA*	RecA-dependent autoproteolyzed regulator
*disA*	–	No	RecA-dependent stress sensor, HJ binding
*dinG*	–	No	3′→5′ ssDNA exo(ribo)nuclease, ATPase ^h^
*rnhC*	–	*rnhA*	5′→3′ exoribonuclease, endoribonuclease
*rnjA*	*ykqC*	No	5′→3′ exoribonuclease
*fenA*	*exoR, ypcP*	*xni*?	Flap 5′ → 3′ exonuclease
*helD*	*yvgS*	(*helD*)^i^	Removes RNAP, ATPase
*ywqA*	–	*rapA*	Backtracks RNAP
*mfd*	–	*mfd*	Transcription-coupled repair, removes RNAP
*topA* ^d^	–	*topA*	ATP-independent type I topoisomerase
*topB*	–	*topB*	ATP-independent type I topoisomerase
*gyrAB* ^d^	–	*gyrAB*	ATP-dependent type II topoisomerase
*parCD* ^d^	–	*parCD*	ATP-dependent type II topoisomerase
*polY1*	*yqjH*	*dinB*	Error-prone DNAP
*polY2*	*yqjW*	*umuCD*	Error-prone DNAP
*dnaE* ^d^ *polA*	*–* –	No *polA*	Error-prone DNAP, RNA primer extension Repair DNAP
*priA* ^d,j^	*–*	*priA*	Replication restart protein, 3′→5′ DNA helicase
*dnaB* ^d^ *–dnaD* ^d^ *–dnaI* ^d^	–	No	Helicase loader

a Gene name.

b Previous and/or alternative name(s) in *B. subtilis*, and mutants that defined them (http://www.subtiwiki.uni-goettingen.de).

c Functional homologs in *E. coli*. No, indicates the absence of the gene in *E. coli*

d Essential genes.

e RadA/Sms, not to be confused with the RadA recombinase of Archaea, is a 5′→3′ DNA helicase, whereas RadA*_Eco_*, which accelerates RecA-dependent strand exchange, lacks DNA helicase activity (Cooper and Lovett 2016).

f PcrA complements the *uvrD_Eco_* defect, but inactivates Rep*_Eco_* (Petit et al. 1998).

g RecU also works as a RecA modulator, whereas RuvC_*Eco*_ only performs HJ resolution.

h DinG lacks the essential FeS domain and exhibits exo(ribo)nuclease activity, whereas DinG*_Eco_* lacks the end-terminal Exo I–III domains and has DNA helicase activity.

i *E. coli helD* gene product is helicase IV, which is a weakly processive 3´ → 5´ DNA helicase with limited sequence identity to HelD, a different domain organization (Larsen et al. 2021), and with no reported role in RNAP removal.

j PriA loads DnaD and DnaB at specific branched structures to reinitiate replication, whereas DnaA is necessary to load DnaD and DnaB proteins at oriC.

When the levels of IR-induced damaged template bases are high or when base excision repair is incomplete, these unrepaired lesions act as roadblocks to the replisome or to the RNAP elongation complex, leading to replicative stress. Survival of reviving spores with damaged template bases was shown to require: (i) the recombinase (RecA); (ii) RecA mediators such as RecO and RecR; (iii) RecA modulators such as RecF, RarA, and RecU; (iv) the LexA regulator; (v) fork remodelers [including RuvAB, RecG, and RadA/Sms (note that RadA is alternatively termed Sms, the gene is termed *radA*)]; (vi) the DNA damage checkpoint sensor and repair licensing factor DisA; (vii) A-family DNAP (as PolI); (viii) Y-family TLS DNAPs (PolY1 and PolY2); and (ix) Mfd, a transcription-repair coupling factor (Table 1) (Vlasic et al. 2014, Raguse et al. 2017, Valenzuela-Garcia et al. 2018). The role of other proteins, as RNase J1, RnhC, FenA, HelD, or RecD2, in the repair of preirradiated spores, remains to be determined. Moreover, certain essential repair-by-recombination proteins (SsbA, PcrA, and PriA) can be inferred to be necessary for spore survival. In the absence of both long-range end resection pathways (as in Δ*addAB* Δ*recJ* cells), however, spores remain recombination proficient and as able to repair DNA damage caused by low IR doses as wt cells (Vlasic et al. 2014). At present, we cannot rule out the possibility that the importance of the RecJ ssDNA 5′→3′ exonuclease in concert with a RecQ-like 3′→5′ DNA helicase (RecQ and RecS) in spore revival may be masked by the existence of redundant pathways.

The programmed expression of proteins ensures that end resection occurs after the completion of the first round of DNA replication, i.e. in the presence of an intact homologous template, beyond 150 min. During the late stage of spore outgrowth and vegetative growth, the expression of nonhomologous end joining enzymes (Ku and LigD) is downregulated, while the expression of the two end resection pathways [AddAB and RecJ–RecQ(RecS)] is upregulated (Mascarenhas et al. 2006, Nicolas et al. 2012, Sinai et al. 2015, Swarge et al. 2020). This way, cells favor the use of error-free homologous recombination pathways and minimize the potentially mutagenic effects of non-homologous end joining during vegetative growth.

## Analysis of the recombination proteins required to rescue a replication stress in vegetative cells

The primary role of the proteins required to mitigate a replication stress should be: (i) to reduce uncoupling of leading and lagging-strand synthesis, limiting the accumulation of ssDNA regions at stalled forks; (ii) to stabilize the stalled replication forks; (iii) to circumvent a lesion *via* different error-free DNA damage tolerance (DDT) subpathways; (iv) to place the lesion on duplex DNA, facilitating its removal by excision repair pathways; (v) to activate error-prone DDT subpathways when a ssDNA region persist; (vi) to underpin replication fork movement; and (vii) to generate a suitable DNA structure for replisome reloading and replication restart. Therefore, we can envision that the aforementioned recombination proteins will participate in one or more of these activities. However, different types of DNA damage or protein roadblocks bound to the template may trigger distinct types of stress that interfere with replication fork progression. Consequently, we cannot rule out that another set of proteins may be required if a different DNA damaging agent or protein roadblock is analyzed.

Many of the proteins (RecA, SsbA, RecO, RecR, RecG, RecX, RuvAB, Mfd, PcrA, PriA, and RadA/Sms) shown to be required to cope with a replication stress are ubiquitous (Table 1). Some other proteins are present in a large number of bacterial species (RecF, RarA, and FenA), while others are less broadly distributed (RecD2, RnhC, DisA, HelD, PolY1, and PolY2) (Table 1). Finally, a set of functions is conserved only within Firmicutes (RecU, RNase J1, and DinG) (Table 1).

### Genetic analyses

The genetic analyses described here have been performed in a background free of mobile genetic elements, as conjugative transposons or prophages, as SPβ and PBSX. In unstressed exponentially growing cells, the absence of the *recA* gene reduces viability by ∼10-fold when cells are grown in rich medium at 37ºC (Sciochetti et al. 2001, Carrasco et al. 2004), suggesting that replication stress occurs even in the absence of exogenous DNA damage, and that the RecA protein is important to rescue it. However, the picture is less evident when other *rec*-deficient strains were analyzed. The single knockout of other genes as *recO, recR, recF, recD2, rarA, radA*, or *dinG* has little to no effect on cell proliferation in rich medium (Sanchez et al. 2005, Romero 2018). Deletion of *recG, recU, ruvAB*, or *rnhC* reduces cell viability by ∼5-fold, with RnhC depletion also conferring a temperature sensitive phenotype (Sanchez et al. 2005, 2007, Fukushima et al. 2007, Gándara et al. 2017, Romero 2018, Schroeder et al. 2023).

Several proteins can perform redundant activities in the cell, and only double mutants uncover the important role of these enzymes during the exponential growth in rich medium in unstressed conditions. It was shown that the deletion of the *rarA* gene severely compromises cell viability in mutants in *recF* (15-fold), *recO* (60-fold), or *recA* (145-fold) (Romero et al. 2020). The reason for this decay in viability remains poorly understood, because *rarA* is epistatic with *recF, recO* and *recA* when cells are damaged by mitomycin C. When the *pcrA* essential gene is translationally fused to a *ssrA* degradation tag, cell viability is reduced by >1000-fold after induction of its degradation (Merrikh et al. 2015). The lethality of depleting the helicase PcrA is partially suppressed by inactivation of *recA, recO, recR*, or *recF*, but not by inactivation of *rarA, recD2, recX, recU, rnhC*, or *dinG* (Petit and Ehrlich 2002, Moreno-Del Alamo et al. 2020, 2021). This suggest that PcrA is essential to prevent the formation of RecA-dependent toxic recombination intermediates.

DNA translocases are crucial to ameliorate a replication stress. Inactivation of *recG* is synthetically lethal in the Δ*ruvAB* context, and RecD2 depletion reduces cell viability by >500-fold in the Δ*ruvAB* or Δ*recG* context, showing that fork remodelers are essential for bacterial growth (Sanchez et al. 2005, Torres et al. 2017).

RTCs lead to pervasive replacement loop (R-loop) formation, and the ribonuclease RnhC is the primary enzyme to remove them (Ohtani et al. 1999, Lang et al. 2017), and RecA plays an essential, albeit poorly understood, role in such process. Inactivation of *rnhC* is synthetically lethal in the Δ*recO* or Δ*recA* context, but not in the Δ*dinG* background (Moreno-Del Alamo et al. 2021), suggesting that RecO and RecA play a crucial, though undefined, role in the resolution of RTCs. RecO and RecA could contribute to the removal of toxic R-loops, as demonstrated for the BRCA2 mediator in eukaryotes (functional counterpart of bacterial RecO) (Bhatia et al. 2014). Furthermore, the Δ*rnhC* Δ*fenA* and Δ*rnhC* Δ*polA* mutant strains are also not viable when grown in LB medium at 25ºC (Randall et al. 2019).

Genetic analyses of cells depleted of a recombination protein show different levels of sensitivity to DNA damaging agents that impede fork progression by producing alkylated bases [as methyl methane sulfonate (MMS)] or helix-distorting lesions (as 4-nitroquiniline 1-oxide [4NQO]): (i) Δ*dinG* cells are moderately sensitive; (ii) Δ*disA*, Δ*radA*, Δ*rarA*, Δ*rnhC*, Δ*recD2*, and PcrA-partially depleted cells are sensitive; (iii) Δ*recR*, Δ*recF*, Δ*ruvAB*, Δ*recG*, and Δ*recU* cells are very sensitive; and (iv) Δ*recO* and Δ*recA* mutants are extremely sensitive (Alonso et al. 1988, 1990, 1991, Fernández et al. 1999, Carrasco et al. 2001, Sanchez et al. 2005, 2007, Cañas et al. 2008, Cárdenas et al. 2012, Raguse et al. 2017, Torres et al. 2017, Moreno-Del Alamo et al. 2021).

Further experiments revealed various genetic interactions among them. First, except for *rnhC*, the remaining genes are epistatic to *recA* in response to MMS- or 4NQO-induced damage (Alonso et al. 1992, 1993a, 2013, Ayora et al. 1996, Carrasco et al. 2001, Cárdenas et al. 2009, 2011, Moreno-Del Alamo et al. 2021). Second, RecO and RecR act prior to RecA (Kidane et al. 2004, Lenhart et al. 2014), and the *recA*73 mutation partially suppresses the Δ*recO* or Δ*recR* phenotype (Alonso and Lüder 1991). The *recO* gene is epistatic to *rarA* or *recF*, but not to *recD*2, *recX* or *recU*, in response to 4NQO- or MMS-induced lesions (Fernández et al. 1999, Cárdenas et al. 2012, Romero 2018, Romero et al. 2020). Third, the DNA repair defect of Δ*recX* or Δ*recD2* cells is partially suppressed by the inactivation of *rarA* (Romero et al. 2019a,b). Fourth, *radA* and *disA* are epistatic to *recG* or *ruvAB*, but not to *recD*2 in response to 4NQO- or MMS-induced lesions (Gándara et al. 2017, Raguse et al. 2017). Finally, *pcrA* is epistatic to *recA, recO*, or *recR*, but it is not epistatic to *recU, recX, recD2, recU, dinG*, or *rnhC* in response to 4NQO- or MMS-induced damage (Petit and Ehrlich 2002, Moreno-Del Alamo et al. 2020, 2021). These findings demonstrate that when the DNA is damaged, multiple recombination proteins contribute to circumvent/bypass the lesion, with RecA playing a central role in this process.

### RecA and its mediators and modulators

The RecA recombinase is the central player in homologous recombination in all bacteria (Cox 2007, Kowalczykowski 2015, Bell and Kowalczykowski 2016). RecA in the ATP bound form (RecA·ATP), nucleates and forms filaments onto ssDNA, and performs homology search and strand exchange only in the presence of its accessory factors, both *in vivo* and *in vitro* (Lovett and Roberts 1985, Carrasco et al. 2015). Those proteins that act before RecA·ATP nucleation are referred to as mediators, and the ones that contribute to RecA filament dynamics and act during homology search and DNA strand exchange are known as modulators.

In *B. subtilis*, the mediators identified so far are RecO–SsbA–RecR, and perhaps RadA/Sms–SsbA. A physical interaction of the SsbA, RecO, and RecR mediators with RecA has not been documented, but RecO interacts with SsbA, and fluorescence microscopy studies revealed that the positive mediators RecO and RecR are necessary for RecA–GFP foci formation (Kidane and Graumann 2005, Costes et al. 2010, Lenhart et al. 2014). *In vitro*, SsbA binds ssDNA with picomolar affinity, and inhibits RecA·ATP nucleation and filament growth on the SsbA–ssDNA complexes (Yadav et al. 2012, 2014). SsbA interacts with and loads RecO onto SsbA-coated ssDNA. RecO is sufficient to partially displace SsbA and facilitate RecA nucleation onto SsbA-coated ssDNA (Carrasco et al. 2015). The concerted action of SsbA and RecO mediators is necessary and sufficient to activate RecA to catalyze plasmid-size DNA strand exchange (Carrasco et al. 2015), but *in vivo* RecR is also required (Fernández et al. 1999). The activity of the RecR mediator is less understood. *In vitro*, RecR binds both dsDNA and ssDNA with similar efficiency and in a cooperative manner (Alonso et al. 1993b). RecR binds to supercoiled DNA at the intersection of two strands, operating as a barrier for the diffusion of relaxed DNA (Ayora et al. 1997). It is believed that *in vivo* it may stabilize DNA regions to facilitate recombination.

The RadA/Sms·ATP enzyme may work as a specialized positive mediator. *In vitro*, RadA/Sms is a 5′→3′ DNA helicase that interacts with and loads RecA onto SsbA-coated ssDNA, but such protein–protein interaction does not activate RecA·ATP to catalyze plasmid-size DNA strand exchange even in the presence of RecO and SsbA (Torres et al. 2023). If RadA/Sms works as a RecA mediator *in vivo* remains to be tested. Finally, unlike RecBCD*_Eco_* (Kowalczykowski 2015), its functional analog in *B. subtilis*, the AddAB helicase–nucleases complex, neither contributes to RecA foci formation nor facilitates RecA nucleation onto SsbA-coated ssDNA (Carrasco et al. 2015, Yeesin 2019).

**Figure 2.**
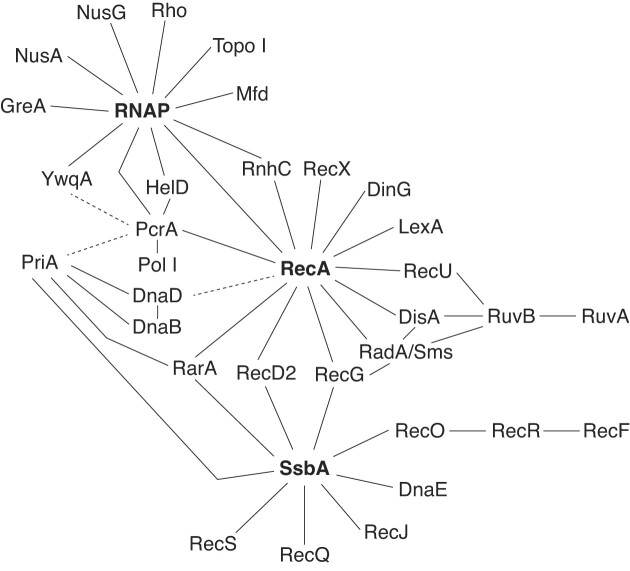
Protein–protein interaction network in *B. subtilis*. RNAP, RecA, and SsbA are protein–protein interaction hubs that may connect several proteins involved in the processing of stalled replication forks. Solid lines show protein–protein interactions proven by pull-downs, bacterial two-hybrid system, and/or confirmed by biochemical or biophysical experiments. The dotted lines show suggested interactions.

The modulators can exert a positive (RecF and RarA) or negative (RecX, RecU, RecD2, and PcrA) regulation on RecA filament growth. Recent live studies have shown that modulators do not impair RecA foci formation, but regulate RecA nucleoprotein filament dynamics (Cárdenas et al. 2012, Lenhart et al. 2014, Romero et al. 2020, Ramos et al. 2022). RarA, RecX, RecU, RecD2, and PcrA physically interact with RecA (Fig. 2), whereas the interaction of RecF with RecA has not been studied. RecF and RarA facilitate the conversion of RecA–GFP foci on threads that are believed to correspond to nucleoprotein filaments (Cárdenas et al. 2012, Romero et al. 2020). SsbA bound to ssDNA interacts with and loads the positive modulator RarA that facilitates RecA filament growth (Carrasco et al. 2018). On the other hand, RarA limits PriA-dependent replication restart *in vitro* (Carrasco et al. 2018). The RecF positive modulator binds dsDNA and ssDNA with similar efficiency (Ayora and Alonso 1997). Both RecF and RarA counteract the negative effect of RecX and RecU on the formation of RecA threads (filaments) (Cárdenas et al. 2012, Lenhart et al. 2014, Romero et al. 2019a). In fact, in the absence of RecF or RarA, the formation of RecA threads is impaired, and thereby SOS induction by LexA self-cleavage, which is aided by the RecA–ssDNA filament (also termed RecA*), is reduced (Gassel and Alonso 1989, Romero et al. 2020). Conversely, in the absence of RecX, RecU, or RecD2, RecA filaments persist for a longer period (Cárdenas et al. 2012, Le et al. 2017, Serrano et al. 2018, Romero et al. 2020, Ramos et al. 2022).

RecX and RecD2 actively disassemble RecA nucleoprotein filaments, while RecU passively facilitates RecA disassembly (Cárdenas et al. 2012, Le et al. 2017, Serrano et al. 2018, Ramos et al. 2022). In addition, RecU forms together with RuvAB the resolvasome, that resolves HJs (Carrasco et al. 2004, 2009, Cañas et al. 2014). The RecD2 helicase, translocating in the 5′→3′ direction, removes RecA bound to the ssDNA region present in collapsed forks to facilitate *in vitro* replication restart (Ramos et al. 2022). Similarly, PcrA translocating in the 3′→5′ direction actively disassembles RecA from ssDNA to prevent unnecessary recombination, with SsbA and RecO counterbalancing such activity (Park et al. 2010, Carrasco et al. 2022).

### Fork remodelers

*Bacillus subtilis* encodes at least eight DNA repair helicases that interact with branched intermediates, either directly [RuvAB, RecG, RecD2, RadA/Sms, PriA, and RecQ-like (RecQ and RecS)] or indirectly (PcrA). Live cell studies have revealed that RecD2, PriA, RecQ, and RecS colocalize with SsbA at replication forks positions, suggesting that these DNA helicases travel with replication forks in unstressed cells (Lecointe et al. 2007, Costes et al. 2010). PriA, PcrA, RecQ, and perhaps its unexplored paralog RecS unwind DNA with 3′→5′ polarity, however, very little is known about their role in fork remodeling (Soultanas et al. 2000, Polard et al. 2002, Qin et al. 2014, Matthews and Simmons 2022). *In vitro*, PriA binds to a variety of DNA substrates, including ssDNA, ssDNA with a single-stranded initiation site (*ssiA*), displacement loops (D-loops), unreplicated forks, partially replicated forks as 5′-fork DNA (a replication fork with a fully synthesized lagging-strand and a gap in the leading-strand), and 3′-fork DNA (a replication fork with a fully synthesized leading-strand and a gap in the lagging-strand) (Marsin et al. 2001, Polard et al. 2002, Lecointe et al. 2007). Upon interaction with SsbA the strong ATPase activity of PriA is inhibited (Polard et al. 2002). PriA removes the nascent lagging-strand of a 5′-fork DNA substrate when SsbA and/or SsbAΔC35 are present (Lecointe et al. 2007), but the unwinding activity of PriA on a 3′-fork DNA substrate has not been tested. Due to conflicting results, it is still unclear whether the PriA motor activity is crucial for replication restart. *In vivo*, PriA, in concert with DnaD, DnaB, and DnaI, loads the replicative DNA helicase DnaC in regions outside *oriC* (see above).

*Bacillus subtilis* encodes two RecQ-like motors, RecQ and RecS (Fernández et al. 1998). RecQ unwinds the template lagging-strand of 3′-fork DNA or fully replicated DNA substrates and disrupts HJ DNA (Qin et al. 2014), but little is known about the activities of RecS. Nevertheless, the contribution of RecQ and RecS upon replication stress is thought to be minor if any, because in the absence of RecQ or RecS reviving spores remain recombination proficient and as capable of repairing DNA damage after low IR doses as wt cells (Vlasic et al. 2014).

PcrA has a template-clearing role at RTCs, but there is no information on whether PcrA works as an accessory replication fork DNA helicase. PcrA physically interacts with and actively displaces RecA from ssDNA (Park et al. 2010, Carrasco et al. 2022). PcrA interacts with and backtracks a paused RNAP, and unwinds the RNA moiety of an R-loop, but it fails to remodel a stalled fork (Soultanas et al. 2000, Delumeau et al. 2011, Sanders et al. 2017, Torres and Alonso 2021, Carrasco et al. 2022). In *E. coli*, three accessory DNA helicases (Rep, UvrD, and DinG), which act at stalled replication forks, remove the protein barriers without fork remodeling (Guy et al. 2009, Boubakri et al. 2010, Hawkins et al. 2019, Syeda et al. 2019). DinG of Firmicutes origin lacks a DNA helicase activity, it contains an extra 245–260 aminoacids long N-terminal region with a DDED exonuclease domain, and shows a 3′→5′ exo(ribo)nuclease activity (McRobbie et al. 2012, Carrasco et al. 2023).

Based on the phenotypes observed for mutants in *ruvAB, recG, recD2*, and *radA* (see above), it is assumed that these helicases remodel the stalled forks to provide a significant replication stress relief, and they may have some redundant activities. The RuvAB complex, composed of the RuvA and RuvB subunits, is a helicase that catalyzes HJ branch migration (Cañas et al. 2014). RuvAB efficiently catalyzes fork restoration after fork reversal, but fails to reverse a stalled fork (Gándara et al. 2021). Upon interaction of RuvAB with RecU, the RuvAB–RecU resolvasome complex is formed (Carrasco et al. 2009, Cañas et al. 2011, 2014). The resolvasome branch migrates reversed forks or HJs formed during DSB repair, and catalyzes HJ resolution when a RecU cognate site is exposed at the junction (Ayora et al. 2004, McGregor et al. 2005, Cañas et al. 2011, 2014, Suzuki et al. 2014).

RecG binds and unwinds a variety of branched DNA substrates, including HJs, and partially replicated 5′- and 3′-forks (Torres et al. 2021), but it might not unwind R-loops (Wen et al. 2005). RecG unwinds stalled forks to reverse them, and regresses a HJ DNA leading to fork restoration (Cañas et al. 2014, Torres et al. 2021). These fork remodeling activities mediated by RecG are believed to contribute to PriA-dependent replication restart.

RecD2 is a 5′→3′ helicase (Walsh et al. 2014) with branch migration activity on three-strand recombination intermediates (D-loops) (Ramos et al. 2022), but its activity on the remodeling of stalled or reversed forks remains elusive. RadA/Sms, which is a ring shaped hexameric 5′→3′ DNA helicase, unwinds DNA in the presence of an available 5′-tail without the need for any accessory protein (Marie et al. 2017, Torres et al. 2019a). Acting as a mediator, RadA/Sms partially displaces SsbA and facilitates RecA nucleation on the 5′-fork DNA or ssDNA (Torres et al. 2019c, 2023, Hertzog et al. 2023). Subsequently, RecA acting as a loader, activates RadA/Sms to unwind several structures, such as mobile D-loops, 5′-fork DNAs, or reversed forks with a nascent leading-strand longer than the nascent lagging-strand (Torres et al. 2019a). The fork clearance activity mediated by RadA/Sms is believed to be important to create the proper substrate for PriA-dependent replication restart.

### DisA contributes to safeguard genome integrity

How is replication stress sensed in *B. subtilis* cells? The DisA checkpoint protein was originally described as a safeguard of genome integrity in *B. subtilis* sporulating cells. DisA scans the chromosome, and delays entry into sporulation in the presence of DNA damage (Bejerano-Sagie et al. 2006). Single-molecule fluorescent imaging cell analysis revealed that in a large majority of unstressed sporulating cells (∼88%), DisA forms a highly dynamic focus that transiently associates with and dissociates from the nucleoid, moving rapidly along the chromosome scanning for “perturbations” (Bejerano-Sagie et al. 2006, Torres et al. 2019c). Transient binding to DNA is required for DisA scanning and pausing, since its variant lacking the RuvA-like DNA binding domain (DisA∆C290) forms a focus that moves freely on the cytosol (Torres et al. 2019c).

While scanning, DisA synthesizes the essential second messenger cyclic 3′, 5′-diadenosine monophosphate (c-di-AMP) (Oppenheimer-Shaanan et al. 2011), being the major c-di-AMP synthase in *B. subtilis* cells (Witte et al. 2008, Gándara and Alonso 2015). In response to DNA damage, DisA pauses, to form a static focus in >95% of sporulating cells, with subsequent suppression of c-di-AMP synthesis, to levels comparable to those of the Δ*disA* strain (Bejerano-Sagie et al. 2006, Oppenheimer-Shaanan et al. 2011, Gándara et al. 2017). These lowered c-di-AMP levels indirectly trigger (p)ppGpp synthesis, which, in turn, reduces the GTP pool (Kruger et al. 2021). (p)ppGpp inhibits DnaG activity (Wang et al. 2007, Giramma et al. 2021, Kruger et al. 2021). *In vitro* DisA does not affect PriA-dependent replication restart, but it slightly increases the length of the Okazaki fragments (Raguse et al. 2017), an effect that has been also observed when DnaG concentrations are lowered (Seco et al. 2013). It is likely that DisA plays a fail–safe mechanism to ensure complete and accurate DNA replication before the cell enters in the sporulation state (Bejerano-Sagie et al. 2006).

To understand which signal(s) are being recognized by and pause DisA movement upon DNA damage, both *in vivo* and *in vitro* experiments were conducted. It was observed that *in vitro* DisA synthesizes c-di-AMP with similar efficiency in presence or absence of dsDNA, but c-di-AMP synthesis is inhibited upon DisA binding to branched intermediates [forks, or 3- and 4-way junctions, which mimic D-loops and HJ structures (reversed forks), respectively] (Witte et al. 2008, Gándara et al. 2017, Torres et al. 2019c). Subsequently, it was tested whether DisA pausing upon DNA damage occurs in the absence of recombination functions *in vivo*. Fluorescent imaging revealed that DisA-GFP foci fail to pause in ∆*recO* or ∆*recA* cells in the presence of MMS-induced lesions (Torres et al. 2019c). On the contrary, when both end resection pathways were inactivated (∆*addAB* ∆*recJ* cells), DisA pausing upon MMS-induced DNA damage was still observed (Torres et al. 2019c). From these findings, it is likely that: (i) the signal recognized by DisA is independent of AddAB- or RecJ-mediated end resection; and (ii) RecA, which may bind to these ssDNA regions that arise upon replication stress by MMS treatment, interacts with and may recruit or pause DisA at stalled or reversed forks (Torres and Alonso 2021).

*disA* forms an operon with *radA* and genes involved in quality control and protein phosphorylation (Gándara and Alonso 2015). Upon damage, DisA pauses its dynamic movement in sporulating ∆*radA* cells as in the wt control (Bejerano-Sagie et al. 2006), suggesting that RadA/Sms acts either after DisA or concomitantly with it (Torres and Alonso 2021). *In vitro*, addition of RadA/Sms to DisA-bound to a branched intermediate blocks c-di-AMP synthesis (Torres et al. 2019b).

DisA-mediated scanning of DNA has also been studied in exponentially growing cells. Single-molecule fluorescent imaging of unstressed exponentially growing wt cells revealed that DisA forms dynamic foci that colocalize with the nucleoid in ∼88% of the cells, while 10%–12% of cells contained spontaneous static foci (Gándara et al. 2017). Exponentially growing cells also contain a similar number of spontaneous RecA foci and the large majority of these foci colocalize with stalled forks (Simmons et al. 2007, Lenhart et al. 2014). *In vitro* studies revealed that DisA interacts with and inhibits RecA-mediated DNA strand exchange (Torres et al. 2019c). This result suggests that at least some repair mechanisms to reactivate stalled forks may not require the strand exchange activity of RecA.

Live cell studies have revealed that RadA/Sms also forms dynamic foci that colocalize with the nucleoid in ∼63% of the cells, but DisA and RadA/Sms foci only transiently colocalize (in ∼27% of cells) (Gándara et al. 2017). In the Δ*recG* and Δ*recU* mutants, in which branched intermediates (stalled or reversed forks) accumulate *in vivo* (Carrasco et al. 2004, Sanchez et al. 2007), DisA-YFP forms static foci that mostly colocalize with the DNA bulk in >90% of cells (Gándara et al. 2017).

*In vitro*, DisA limits the activity of many proteins that act at the stalled fork. DisA bound to stalled or reversed forks inhibits fork remodeling by RuvAB and RecG, DNA unwinding by RadA/Sms, and RecU-mediated resolution of HJ structures (Gándara et al. 2021, Torres and Alonso 2021, Torres et al. 2021, 2023). However, DisA bound to a branched intermediate neither affects PriA-dependent replication restart (Raguse et al. 2017) nor PcrA-mediated DNA unwinding (Torres et al. 2021), suggesting that the inhibitory activity of DisA over some recombination proteins is protein specific.

All these analyses suggest that upon replication stress branched intermediates accumulate, inducing DisA pausing, blocking c-di-AMP synthesis, and thereby indirectly inhibiting DnaG (Wang et al. 2007, Gándara et al. 2017). Consequently, DisA pausing might decrease the overall velocity of the sister replisome, perhaps to coordinate the clockwise and counterclockwise replisomes and allow time for DNA repair (Gándara and Alonso 2015, Gándara et al. 2017).

### Interactome of proteins that act at stalled forks or RTCs

The physical interaction among the proteins that show a genetic and biochemical interplay upon replication stress has been analyzed using pull-downs or a bacterial two-hybrid system *in vivo* and some of them have been confirmed through different *in vitro* protein–protein interaction assays (Fig. 2). These analyses show a dense interconnexion between many proteins, highlighting the importance of their coordination for the repair of stalled forks. Several discrete hubs were observed. First, SsbA interacts with RecG, RecO, RecD2, RarA, PriA, DnaE, RecQ, and RecS among others, and many of these proteins appear to travel with the replisome during unperturbed replication (Lecointe et al. 2007, Costes et al. 2010).

Second, RecA interacts with DisA, RadA/Sms, RecG, LexA, RecU, RecX, RarA, RecD2, PcrA, RNAP, RnhC, and DinG (Carrasco et al. 2005, 2022, 2023, Groban et al. 2005, Cañas et al. 2008, Torres et al. 2019a, c, Ramos et al. 2022). Among these protein interactions, it can be highlighted that RarA, RecD2, and DnaE are part of both SsbA and RecA interactomes, and that RarA physically interacts with PriA (Carrasco et al. 2018). Similarly, the preprimosomal proteins interact among themselves (Marsin et al. 2001, Polard et al. 2002, Smits et al. 2011), and indirectly with RecA (Million-Weaver et al. 2015).

Third, PcrA interacts with RNAP, RecA, PriA, HelD, and PolI (also known as PolA), among others (Sanders et al. 2017).

Fourth, RadA/Sms physically interacts with RecG, RecA, DisA, and RuvAB (Gándara et al. 2021, Torres and Alonso 2021, Torres et al. 2021). DisA physically interacts with RecA, RadA/Sms, and with the RuvB subunit of the RuvAB complex (Torres et al. 2019b, Gándara et al. 2021). RecG establishes direct cross-talk with the RecA and SsbA hubs. Similarly, RecU is indirectly part of the RecA and DisA interactomes, because it directly interacts with RecA and RuvB, which interacts with DisA (Fig. 2) (Carrasco et al. 2005, 2009, Cañas et al. 2008, 2011).

Finally, RNAP interacts with HelD, Mfd, YwqA (homolog to RapA*_Eco_*), RecA, PcrA, RnhC, GreA (Fig. 2), as well with NusA, NusG, Rho, and TopA (also termed Topo I) (Delumeau et al. 2011). Remarkably, RnhC interacts with RNAP even in the absence of exogenous DNA damage, highlighting the importance of the resolution of RTCs (Delumeau et al. 2011). Furthermore, certain functions of the translation complex interact with DinG (Costes et al. 2010). Among these protein interactions, it can be highlighted that PcrA and RnhC are part of RecA and RNAP protein-interaction hubs (Fig. 2).

## Single molecule analyses show the dynamics of replisomes and recombination proteins during exponential growth

Single-molecule imaging of fluorescently labelled proteins has uncovered the dynamic behavior of replisomes in exponentially growing *B. subtilis* cells, where transient meropolyploidy and multiple repair pathways and recombination intermediates coexist. Recent live cell studies have revealed that the average residence time of PolC, DnaE, and DnaX in cells grown in minimal media is short (time-scale of seconds) (Liao et al. 2016, Hernández-Tamayo et al. 2019), and significant variations (from ∼8 min to <2 s) have been reported for the residence time of DnaC (Mangiameli et al. 2017, Hernández-Tamayo et al. 2021).

Stoichiometric analyses of DnaC, DnaX, and PolC revealed that ∼45% of unstressed cells have only one replisome per cell, suggesting that the other one has been disassembled upon replication stress (Mangiameli et al. 2017). It was estimated that roughly five replisome disassembly events occur per cell cycle, due to clashes with unremoved endogenous threats or with codirectional (CD) RNAPs transcribing highly expressed genes (Mangiameli et al. 2017). The high frequency of RTCs is further supported from the following observations: (i) when replication restart is impeded by PriA depletion, the number of unstressed cells having two DnaC foci (i.e. replisomes loaded at *oriC* and not disassembled) is significantly reduced, to ∼13% of total cells (Mangiameli et al. 2017); (ii) the replisome undergoes transient locus-specific pausing at ribosomal RNA loci in exponentially growing unstressed *B. subtilis* wt cells (Huang et al. 2023); and (iii) transcription inhibition by treatment with rifampicin increases replisome lifetime as well as the rate of replication, and prevents RecA foci formation (Mangiameli et al. 2017, Yeesin 2019).

To avoid replication fork collapse and ultimately maintain genome stability, stalling impediments must be repaired, circumvented, or bypassed efficiently before replisome reloading. RecA–GFP, expressed from its native locus and under the control of its native promoter, is largely cytosolic in unstressed cells (Simmons et al. 2007). However, several reports have shown that ∼15% of total unstressed cells contain RecA foci that colocalize with the nucleoid, and the large majority of these foci are either at midcell or at quarter-cell positions, as the replisome (>85% colocalization with DnaX) (Simmons et al. 2007, Wang et al. 2007). ChIP-Seq analyses showed RecA accumulation at sites of engineered RTCs (Million-Weaver et al. 2015). Furthermore, the preprimosomal proteins DnaD and DnaB are associated with *rrn* loci (Merrikh et al. 2011), and RecA contributes to DnaD association at sites of RTCs (Million-Weaver et al. 2015). Thus, it is tempting to speculate that in the absence of external damage, when cells are grown exponentially in rich medium, RecA accumulates at sites where fork progression is impeded, i.e. where the replisome clashes with CD arrays of RNAP at highly transcribed gene clusters, such as the *rrn* operons, or at sites with HO conflicts, like artificially inversed rRNA loci.

Upon an inhibition of replisome assembly at *oriC*, induced by DnaA and DnaN depletion, the percentage of cells with RecA foci correlates with the percentage of cells that contain active replisomes, suggesting that the formation of RecA foci requires ongoing DNA replication (Simmons et al. 2007). Another study showed that ∼15% of exponentially growing unstressed cells have RecA–GFP foci, but the SOS response, which is induced by the autocleavage of the transcriptional repressor LexA facilitated by RecA* (Sassanfar and Roberts 1990), is only induced in <0.5% of total cells (Simmons et al. 2007, 2009). This suggests that RecA foci formation is necessary but not sufficient for SOS induction.

RecO, RecF, and RarA also form foci that colocalize with the replisome in the majority of unstressed cells (>85% colocalization) (Costes et al. 2010, Romero et al. 2019a). The positive RecO and RecR mediators are required for RecA–GFP foci formation, but the RecF or RarA positive modulators are not when cells are untreated, but they may contribute in the presence of DNA damage (Gassel and Alonso 1989, Kidane et al. 2005, Lenhart et al. 2014, Romero et al. 2020). Since a high proportion of RecA foci colocalize with the replisome, in the absence of DNA damage, it can be assumed that endogenous barriers on both leading- and lagging-strand templates transiently inhibit fork progression, leading to replisome disassembly, and the formation of lesion-containing gaps (Mangiameli et al. 2017, Yeesin 2019, Stoy et al. 2023). In addition, GFP-RecO is targeted to active replication forks by its interaction with SsbA (Costes et al. 2010). Here, error-free DDT subpathways can contribute to fork stabilization and replication restart in a RecA-dependent manner (including fork reversal, template switching, and lesion bypass).

In *E. coli* cells, however, unremoved endogenous lesions halt PolIII*_Eco_*, but DnaB*_Eco_* continues to unwind dsDNA, albeit at a significantly reduced rate and with no apparent disassembly (Graham et al. 2017). In fact, PriA*_Eco_* foci formation was observed in only ∼7% of total unperturbed exponentially growing cells (Soubry et al. 2019). Single-cell analyses revealed that RecA*_Eco_* is present in storage structures in the vast majority of unperturbed growing cells (∼80%), and that in the remaining cells (∼20%) RecA*_Eco_* disassembles from these storage structures and forms foci. However, these RecA foci scarcely colocalize with DnaX*_Eco_* (∼24% of colocalization) (Ghodke et al. 2019). RecO*_Eco_* and RecR*_Eco_* also form foci that rarely colocalize with PolIII*_Eco_* (Henrikus et al. 2019). In contrast, RecF*_Eco_*, which interacts with DnaN*_Eco_* and with DnaG*_Eco_*, colocalizes with the replisome (Henrikus et al. 2019, Henry et al. 2023). It has been proposed that the RecF_*Eco*_–replisome interaction may destabilize the replisome, which is subsequently reengaged upon DnaG-mediated repriming (Fig. 1A) (Henry et al. 2023). All these results suggest that in *E. coli* the PolIII holoenzyme usually skips over the lesion to leave behind a lesion containing gap, which is mainly processed by an error-free DDT pathway (template switching) (Fig. 1A and B). In contrast, the data obtained in *B. subtilis* suggest that replisome disassembly and RecA dependent replication restart is the main mechanism to rescue a replication stress.

May these recombination *B. subtilis* proteins alter replisome dynamics? *In vitro* two set of activities performed by recombination proteins during replication reinitiation have been observed: those that directly inhibit replication reinitiation, and those that indirectly impair replication elongation. Within the first activity group, RecA is included. *In vitro* RecA, with the help of RecO and SsbA, inhibits PriA-dependent replication reinitiation from a DNA substrate that mimics a 3′-fork DNA (Vlasic et al. 2014). RecD2, which promotes RecA disassembly from ssDNA, plays a dual role *in vitro*: it antagonizes the negative effect exerted by RecA on PriA-dependent DNA replication restart, but at high concentrations inhibits DNA replication restart (Ramos et al. 2022). RarA, at a 3′-fork DNA substrate, also inhibits PriA-dependent replication initiation (Carrasco et al. 2018). Notably, all these effects are at the restart step, because *in vitro* DNA replication elongation remains unaffected by RecA, RecO, RarA, or RecD2 (Vlasic et al. 2014, Carrasco et al. 2018, Ramos et al. 2022). The second activity is performed by DisA, and it is related to the fact that *in vivo*, low levels of c-di-AMP, upon DisA binding to a branched intermediate, indirectly inhibit the DnaG activity (see above) (Wang et al. 2007).

## Different responses to replication stresses

In eukaryotes, replicative stress induces various post-translational modifications, particularly phosphorylation cascades that play critical roles in orchestrating the DNA damage response (Ciccia and Elledge 2010, Zeman and Cimprich 2014, Gaillard and Aguilera 2016, Saxena and Zou 2022). In *B. subtilis*, a fraction of RecA and SsbA is phosphorylated (Elsholz et al. 2012, Schmidt et al. 2014). However, the physiological role of protein phosphorylation in response to replication stress remains largely unexplored, with only few descriptions in the literature (Elsholz et al. 2012, Yadav et al. 2012, Bidnenko et al. 2013). The current hypothesis is that the role of phosphorylation may be to target damaged forms of proteins (Trentini et al. 2016, Gangwal et al. 2023).

In *B. subtilis*, the application of different impediments to replication fork progression has been used to analyze the responses to a replication stress. First, transient arrest of only one replisome (for 90 min), by repressors binding to a discrete operator array located specifically at one arm of the replisome, leaving replication of the other arm and replication reinitiation unaffected, was assayed. Under this condition, virtually all cells experienced a replication roadblock, resulting in altered nucleoid organization, blocked cell division, and leads to the formation of RecA foci in >80% of total cells (Bernard et al. 2010). These RecA foci were not sufficient to trigger the SOS response (Bernard et al. 2010). This suggests that under this specific condition RecA filament growth is downregulated at the stalled fork.

Secondly, DNA replication was inhibited for 40–80 min by HPUra, which blocks elongation of both replisomes by poisoning the PolC subunit of the replicative PolC holoenzyme, (Wang et al. 2007, Bernard et al. 2010). Here, PolC decouples from DnaC, leading to persistent ssDNA regions and to the induction of global stress responses (LexA-dependent and LexA-independent) (Goranov et al. 2006). RecA forms foci in ∼95% of cells (Wang et al. 2007, Bernard et al. 2010). The TLS Y-family DNAP PolY1, which is not induced by DNA damage, in concert with the A-family Pol I, may replace the HPUra-blocked PolC core enzyme to catalyze nucleotide incorporation, which is followed by gap sealing (Fig. 1D and E) (Sung et al. 2003, Duigou et al. 2004, 2005). This poorly understood pathway may be mutagenic. Recent studies have shown that PolY1, which interacts with the DnaN-sliding clamp (Duigou et al. 2005, Timinskas and Venclovas 2019), enriches at or near sites of replication in the absence of DNA damage and colocalizes with DnaX (Marrin et al. 2023).

Thirdly, UV-induced DNA damage, which at low dose produces replication fork stalling and at high doses also replication fork collapse, revealed a more complex response. At low doses (1 J/m^2^), UV light produces ∼40 adducts/chromosome, that can be specifically repaired by nucleotide excision repair (see Courcelle et al. 2006). However, if unrepaired, PolC would encounter these lesions and halt, leading to pervasive replisome disassembly. In fact, the average residence time of DnaC, PolC, DnaE, and DnaX is significantly shortened in response to DNA damage and/or PolC inhibition (Liao et al. 2016, Hernández-Tamayo et al. 2019). In this scenario, RecA formed foci in ∼85% of total cells as early as 5 min after exposure to UV light, and these RecA foci colocalized with DnaX (∼90% colocalization) (Simmons et al. 2007). Despite this, >95% of total irradiated cells form colonies, the level of RecA protein does not significantly increase compared to unstressed cells, and there is poor or no SOS induction (Simmons et al. 2007). Thus, it can be envisioned that: (i) at low UV doses (1 J/m^2^), DNA lesions at both leading- and lagging-strands block fork progression, leading to pervasive replisome disassembly, and then RecA, with the help of mediators, assembles at the ssDNA region of the stalled fork; and (ii) RecA forms foci, perhaps to protect the stalled fork, rather than threads that would contribute to homology search and DNA strand invasion as well as to SOS induction. Although it cannot be ruled out that a blocking lesion on the lagging-strand can be potentially skipped by priming a new Okazaki fragment, we consider that the high proportion of RecA foci colocalizing with replisomes does not support such assumption (Simmons et al. 2007, Wang et al. 2007, Lenhart et al. 2014).

At 40 J/m^2^ (∼1600 adducts/chromosome), RecA forms foci that colocalize with DnaX (∼84% colocalization) in ∼97% of the cells (Lenhart et al. 2014). At an intermediate UV dose (25 J/m^2^), these RecA foci are also observed. The formation of these RecA foci is strictly dependent on RecO and RecR, but not on RecF, which assembles later at repair centers upon DNA damage (Kidane et al. 2004, Lenhart et al. 2014). These RecA foci are then converted into dynamic threads (RecA nucleoprotein filaments). In the absence of RecF, the percentage of cells with RecA foci decreased, whereas in the absence of RecD2, the percentage of cells with RecA foci and threads significantly increased (Lenhart et al. 2014, Walsh et al. 2014). RecA threads are short-lived in *recF*15 or Δ*rarA* cells, but long-lived in Δ*recD2*, Δ*recX*, and Δ*recU* cells (Kidane et al. 2009, Cárdenas et al. 2012, Carrasco et al. 2018, Romero et al. 2020, Ramos et al. 2022). It is likely that RecA filament extension (RecA threads) is a constrained step regulated by positive and negative modulators.

A RecA nucleoprotein filament causes LexA self-cleavage and SOS induction (Au et al. 2005). Upon SOS induction, in a LexA- and RecA-dependent manner, 31–33 genes undergo a change in expression, but only 8 (*recA, lexA, ruvAB, uvrBA, pcrA*, and *polY2*) of these genes are shared with *E. coli* (Au et al. 2005).

In response to DNA damage, there are also RecA-independent responses to replication stress, but they are poorly understood (Goranov et al. 2006). There is also a global DnaA-dependent transcriptional response elicited by replicative stress that alters the expression of >100 genes (including essential replication genes) (Goranov et al. 2005). Finally, there is a response mediated by the stress-associated transcription factor σ^M^, which alters the expression of ∼57 genes, including *disA* and *recU* (Eiamphungporn and Helmann 2008, Carrasco et al. 2009).

## Does *B. subtilis* RecA exhibit a noncanonical activity?

The bacterial (RecA) and mammalian (Rad51) recombinase play their prime role in homologous recombination through strand invasion and DNA strand exchange (Cox 2007, Kowalczykowski 2015, Bell and Kowalczykowski 2016). In mammals, efficient fork reversal requires Rad51, although its enzymatic activity is not required (Betous et al. 2013, Neelsen and Lopes 2015, Zellweger et al. 2015). This apparent paradox might be explained by the existence of two distinct Rad51 activities: canonical and noncanonical (Zellweger et al. 2015). The canonical Rad51 activities include DNA strand invasion and strand exchange, whereas its noncanonical activity contributes to overcome a replication stress by protecting a reversed fork independently of its catalytic functions (Thomas et al. 2023). Using a similar nomenclature, we aim to define both activities for RecA.

In a canonical activity, RecA·ATP cooperatively binds with high affinity to ssDNA, forming helical nucleoprotein filaments. ATP hydrolysis throughout the filament leads to RecA·ADP, which predominantly dissociates of the ssDNA from the filament ends. The ATPase activity of RecA is not required for the key function of homology search, but it is essential for extensive (plasmid-size) DNA strand exchange, and for bypassing structural barriers in the DNA substrates (Cox 2007, Bell and Kowalczykowski 2016). The canonical RecA·ATP activities have been well-documented over the years (reviewed in Cox 2007, Bell and Kowalczykowski 2016).

The nonrecombinogenic or noncanonical RecA activities refer to those activities that are independent of strand invasion and DNA strand exchange. These activities remain largely elusive since they are difficult to prove in live cells. However, based on available information, we propose that in the absence of ATP hydrolysis, RecA may protect the fork from degradation, as it has been observed for Rad51 (Zellweger et al. 2015), and help to recruit other proteins for fork processing. There are few pieces of information that indirectly suggest a noncanonical activity of RecA in *B. subtilis*. First, RecA forms foci that are not converted into threads at RTCs or in response to a low UV dose (1 J/m^2^) (Simmons et al. 2007, Million-Weaver et al. 2015). Second, RecA interacts with many proteins, among them with DisA or RadA/Sms, which inhibit the ATPase and the DNA strand exchange activities of RecA, perhaps to prevent RecA from engaging in unnecessary homology search and strand exchange (Torres et al. 2019a, c, Carrasco et al. 2022, 2023) Third, upon artificial inversion of the *rrnIHG* operons, that strongly compromises (>1000-fold) the plating efficiency in LB medium (Srivatsan et al. 2010, Huang et al. 2023), PcrA overexpression improves viability of *recA*^+^ cells, but not of Δ*recA* cells. Contrarily, upon *rrnIHG* inversion, overexpression of RnhC does not improve the viability of *recA*^+^ cells grown on LB, but slightly increases viability of Δ*recA* cells (Yeesin 2019). Thus, it is likely that a noncanonical RecA activity cooperates with PcrA to overcome HO RTCs, and with RnhC to remove R-loops. Finally, RecA promotes swarming motility, and such effect does not require canonical RecA activities (Gomez-Gomez et al. 2007).

Whether these noncanonical activities can be also performed by other bacterial RecA homologs is unknown. In *B. subtilis*, RecA foci formation at locations distal from replisomes is rarely observed (Simmons et al. 2007). Noncanonical RecA activities may protect stalled forks from degradation, and may contribute to suppressing the uncoupling of ongoing replication forks to limit ssDNA accumulation at stalled forks.

## Fork remodeling pathways at stalled forks

All the information reviewed within the previous sections suggests that replication stress in *B. subtilis*, often causes replisome disassembly, and that the repair, modulated by RecA, is spatially and temporarily coupled with DNA replication. Consequently, lesion skipping and postreplicational repair of the gap left behind (Fig. 1A–C) are likely not the primary mechanisms employed to overcome transient stalled replisomes in *B. subtilis*. This coupling between replication and repair would limit ssDNA formation, and indirectly protect the stalled or reversed fork. The nature of the replication stress is a key determinant of the chosen pathway. PolC holoenzyme replacement by either PolY1 or PolY2, should be a minor pathway (Fig. 1D and E), because it could be mutagenic (Aliotta et al. 1996, Sung et al. 2003, Duigou et al. 2004, 2005). It is tempting to speculate that the noncanonical activity of RecA (see above) licenses fork remodeling, serving as the primary avenue to cope with replication stress. Direct *in vivo* documentation of how fork remodeling occurs, which has proven difficult to obtain, is not available. Thus, the present evidence of this process relies on *in vitro* biochemical analyses.

**Figure 3.**
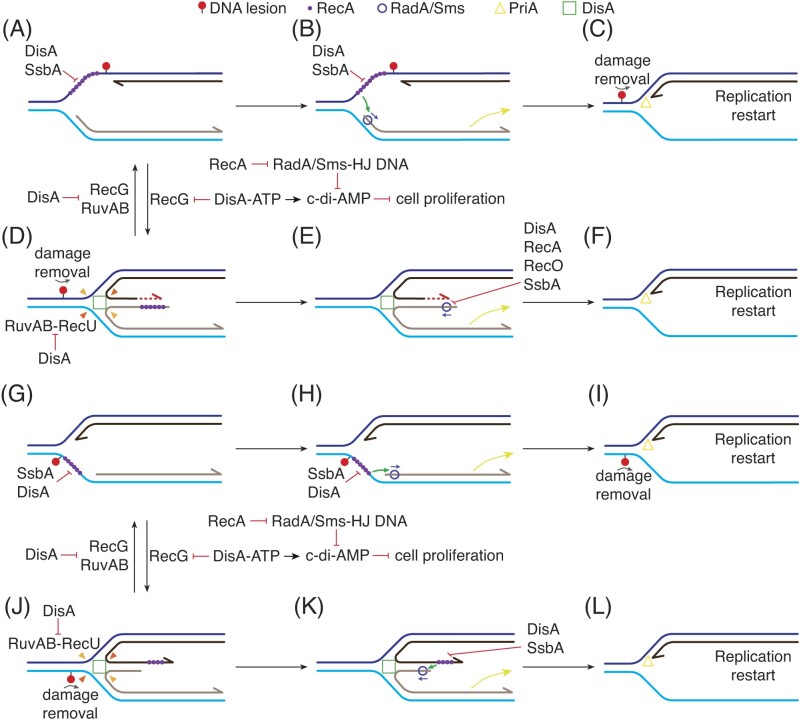
Proposed model for remodeling stalled forks in *B. subtilis*. (A) and (G) When a replisome encounters a lesion in the template strand, it stalls and disassembles. SsbA bound to the resulting lesion-containing gap on the template leading-strand [termed here forked-Lead (A)] or on the template lagging-strand [termed here forked-Lag (G)] inhibits RecA loading. Mediators such as RecO (or RecO–RecR, not depicted), or RadA/Sms, displace SsbA, and interact with and recruit RecA, which then binds onto the lesion-containing gap on the template strand. DisA scans the genome, searching for branched intermediates, and pauses. DisA interacts with and inhibits the ATPase of RecA, and this indirectly avoids filament growth and SOS induction. (B) and (H) RecA bound to the template strand interacts with and loads the RadA/Sms helicase on the nascent-lagging-strand, with RadA/Sms unwinding it. (C) and (I) Spontaneous remodeling (or fork remodeler-mediated) places the deleterious lesion on duplex DNA for its removal by specialized pathways. Finally, PriA, which recognizes a 3′-fork DNA, recruits other preprimosomal proteins (DnaD–DnaD–DnaI) to load the DnaC helicase for replication restart. (D) and (J). Alternatively, the RecG remodeler converts forked-Lead (A) into a HJ DNA with a nascent lagging-strand longer than the leading-strand (termed here HJ-Lag DNA) (D), or the forked-Lag (G) into a HJ DNA with a nascent leading-strand longer than the lagging-strand (termed here HJ-Lead DNA) (J). DisA bound to these HJ structures limits RecG or RuvAB mediated branch migration, and RuvAB–RecU-mediated HJ cleavage. RadA/Sms bound itself (E), or been recruited by RecA bound to HJ-Lead DNA (K), unwinds the nascent lagging-strand to yield a 3′-fork DNA. Then, PriA bound to the 3′-fork DNA substrate recruits other preprimosomal components to reinitiate DNA replication (F) and (L). (E) RecA, with the help of its accessory proteins (RecO and SsbA) or DisA may limit RadA/Sms loading at the 5′-tailed HJ-Lag DNA to facilitate that DNA synthesis occurs by the extension of the nascent leading-strand using the nascent lagging-strand as a template to bypass the deleterious lesion. (K) DisA and SsbA may regulate RadA/Sms recruitment by RecA to HJ-Lead DNA.

Biochemical assays suggest four discrete scenarios to describe how fork remodeling may occur. In the first scenario, the leading-strand replisome stalls by a lesion on the template leading-strand, and replisome disassembly occurs (Fig. 3A, forked-Lead structure). SsbA then binds to the ssDNA gap, but RecA·ATP cannot nucleate on the SsbA–ssDNA complexes (Carrasco et al. 2015). There are two set of mediators that can act at this step. In the first, RecA is loaded at the stalled fork through the joint action of SsbA and RecO (or RecO and RecR *in vivo*). This may facilitate the canonical activities of RecA: RecA nucleation, stimulation of RecA ATPase, and subsequent filament growth and strand invasion, at least *in vitro* (Carrasco et al. 2008, 2015). These canonical RecA activities would promote template switching, and have been previously described in Fig. 1(B) and (C). Alternatively, RadA/Sms partially displaces SsbA from the lesion-containing gap, interacts with and loads RecA, with RadA/Sms inhibiting the RecA ATPase and DNA strand exchange activities (Torres et al. 2023). Additionally, DisA further antagonizes RecA filament growth and DNA strand exchange (Torres et al. 2019c), favoring noncanonical activities of RecA. Then, RecA could load RadA/Sms onto the stalled fork, and this protein may unwind the nascent lagging-strand in the 5′ → 3′ direction (Fig. 3A and B) (Torres et al. 2019a), creating a 3′-fork DNA, the proper structure for PriA-mediated replication restart (Fig. 3B and C). Alternatively, PriA is loaded there with the help of SsbA, and would remove the nascent lagging-strand (Lecointe et al. 2007). In this model, we assume that concomitantly, spontaneous or RecG-mediated fork remodeling occurs, in order to relocate the deleterious lesion into a duplex DNA region, for its removal through specialized excision repair pathways. We cannot rule out that in certain mutant backgrounds a RecQ or RecS enzyme, in concert with RecJ, could displace and degrade the nascent lagging-strand of a stalled or reversed fork, generating a 3′-fork DNA substrate, or that the RecD2 5′→3′ helicase removes it. *In vivo* evidences show that these helicases travel with replication forks (Costes et al. 2010), but whether they perform this activity remains to be tested *in vitro*.

In the second scenario, at the lesion on the template leading-strand of the forked-Lead structure, the RecG fork remodeler, that also travels with the replication fork through its interaction with SsbA (see above), acts at the stalled fork. The RecG translocase reverses the stalled fork into a HJ structure, with pairing of the nascent strands (Fig. 3A–D). This process results in an intermediate where the nascent lagging-strand is longer than the nascent leading-strand (termed here as a HJ-Lag structure), with the deleterious lesion placed on duplex DNA for its removal, and with RecA protecting the extruded ssDNA end. Here, RecA could protect the extruded ssDNA end of the reversed fork, interact with and load DisA (Torres et al. 2019c). DisA bound to the HJ DNA may limit RecG-mediated fork reversal. Simultaneously, RecG bound to HJ DNA may block DisA-mediated c-di-AMP synthesis, indirectly inhibiting DnaG activity (Torres et al. 2021). The RuvAB branch migration translocase, which acts on HJs (Cañas et al. 2014), cannot convert a stalled fork into a reversed fork but can further branch migrate a fork reversed by RecG (Gándara et al. 2021). RuvAB-mediated branch migration may expose the target site for the RuvAB–RecU HJ resolvasome complex. DisA, however, interacts with RuvB and limits RuvAB branch migration and also limits RecU-mediated HJ cleavage (Gándara et al. 2021). This mechanism would prevent the generation of a one-ended DSB.

The nascent leading-strand of the HJ-Lag intermediate may prime DNA synthesis, using the intact nascent lagging-strand as a template. Upon damage removal, there are several proteins that can reconstitute a replication fork. First, the RuvAB or RecG remodelers could catalyze fork restoration (also known as fork regression) (Fig. 3A–D) (Cañas et al. 2014, Gándara et al. 2021, Torres et al. 2021). Alternatively, RadA/Sms bound to the 5′-end of the nascent lagging-strand unwinds it to generate a 3′-fork DNA substrate, a process limited by RecA, SsbA, RecO, or DisA (Fig. 3E and F) (Torres et al. 2023). RecD2 could also perform this activity, but it has not been tested.

In the third scenario (Fig. 3G, forked-Lag structure), a barrier at the template lagging-strand leads to PolC holoenzyme disassembly and SsbA binding to the ssDNA gap. Although PriA could potentially bind to this structure to allow replication restart, it has been observed that a preformed SsbA–ssDNA complex, containing or not a *ssiA* site, significantly inhibits the ATPase activity of PriA (Polard et al. 2002). Here, mediators could facilitate SsbA partial displacement and RecA loading. RecA bound to the template lagging-strand would activate RadA/Sms to bind and unwind the nascent lagging-strand, generating a 3′-fork DNA substrate (Fig. 3G–I). Concomitantly, as in the first scenario, spontaneous or RecG-mediated fork remodeling relocates the lesion to duplex DNA for its removal by specialized repair pathways.

In the fourth scenario, after replisome stalling and disassembly by encountering a damage in the lagging-strand, SsbA (or RecA) interacts with and loads the RecG remodeler at a stalled fork, that reverses it into a HJ-like structure (Fig. 3G–J), as described in the second scenario. This results in an intermediate where the nascent leading-strand is longer that the nascent lagging-strand (termed here HJ-Lead structure), and the deleterious lesion is located on duplex DNA for its removal. As in the second scenario, DisA bound to the HJ-Lead DNA limits the activities of RecG and RuvAB–RecU to prevent the generation of a one-ended DSB (Gándara et al. 2021). RecG blocks DisA-mediated c-di-AMP synthesis, and indirectly could inhibit DnaG activity (Fig. 3J) (Torres et al. 2021). At the HJ-Lead DNA, RecA bound to the longer nascent leading-strand loads RadA/Sms onto the nascent lagging-strand. Subsequently, RadA/Sms unwinds the nascent lagging-strand to create the 3′-fork DNA substrate, in a reaction controlled by SsbA and DisA (Fig. 3K and L) (Torres et al. 2023).

In all scenarios, PriA loading at the resulting 3′-fork structure is mediated by its interaction with SsbA bound to the template lagging-strand (Fig. 3C, F, I, and L). Then, the preprimosome PriA–DnaD–DnaB complex, along with the DnaI chaperone, loads DnaC (Bruand et al. 2001, Marsin et al. 2001, Polard et al. 2002, Velten et al. 2003, Bruand et al. 2005, Smits et al. 2011, Arias-Palomo et al. 2013). Finally, DnaC and SsbA, acting as protein-interaction hubs, recruit the remaining components of the replisome to enable replication restart (Haroniti et al. 2003, Bailey et al. 2007, Rannou et al. 2013).

## Replication fork processing at replication–transcription barriers

As transcription and replication occur simultaneously in bacteria using the same DNA as template, RTCs often occur in actively replicating bacteria (Mirkin and Mirkin 2005, Gaillard and Aguilera 2016, Berti et al. 2020). Transcription–translation coupling, which is thought to reduce RNAP pausing to ensure mRNA synthesis, may not occur in *B. subtilis* (Wang and Artsimovitch 2021). First, live cell studies have revealed that RNAP resides principally within the nucleoid whereas ribosomes are localized almost exclusively outside the nucleoid in *B. subtilis* cells (Lewis et al. 2000). Second, transcription is ∼2-fold faster than translation, suggesting that RNAP outpaces the pioneering ribosome (Johnson et al. 2020). Furthermore, single molecule experiments have revealed that translation occurs in close proximity to the cell poles (Stoll et al. 2022). The spatial separation of transcription and translation in *B. subtilis* may render ribosome-free nascent mRNAs prone to forming hairpins and R-loops, and may facilitate RNAP backtracking (Johnson et al. 2020, Wang and Artsimovitch 2021).

In *B. subtilis* the highly transcribed genes have a CD bias with respect to the moving replisome (Merrikh et al. 2011). *Bacillus subtilis* encodes for 10 *rrn* operons per genome that are CD transcribed with respect to the replisome (Merrikh et al. 2012). The high expression of *rrn* operons leads to certain degree of CD RTCs, fork pausing, and a slight effect (1%–3% of death) in cells growing in a rich medium as LB (Merrikh et al. 2011, Huang et al. 2023). Deletion of nine *rrn* operons results in oversaturation of the single CD *rrnA* locus located at the *oriC* region, and while cells continue to grow, they exhibit a strong increase in R-loop accumulation, longer lag-phases and doubling times than wt cells, and up to ∼12% of cell death when grown in LB medium (Fleurier et al. 2022). A more severe defect is observed when the highly transcribed *rrnIHG* operons are artificially inverted, leading to a HO RTC. Transcription of the *rrn* loci occurs more frequently in rich medium (LB) than in minimal medium. The high transcription levels of the inverted *rrnIHG* operons caused ∼25% of death when cells were plated in minimal medium, and stronger growth defects (∼2000-fold reduction in plating efficiency) when plated in LB medium (Srivatsan et al. 2010, Huang et al. 2023).

The *B. subtilis* RNAP elongation complex is composed of six different subunits (${\alpha_2}\beta{\beta^{\prime}}\omega\delta\epsilon$), with the small $\delta$ and $\epsilon$ subunits, influencing RNAP recycling, only present in bacteria of the Firmicutes phylum (Lane and Darst 2010). Due to its abundance, RNAP elongation complexes are more prone to encountering barriers (such as roadblocks, or DNA lesions) than the replisome (Merrikh et al. 2012, Lang and Merrikh 2018). During transcriptional stress, the stalled RNAP, acting as the primary sensor of the barrier, may recruit the proteins required to ameliorate a potential RTC. Indeed, many proteins predicted to participate in the resolution of CD RTCs interact with RNAP, as PcrA, Mfd, YwqA, HelD, RnhC, GreA, NusA, NusG, Rho, Topo I, and RecA (Fig. 2) (Delumeau et al. 2011, Sanders et al. 2017). Other proteins might be recruited through indirect interaction, *via* RecA (as RecG, RecD2, PcrA, RarA, DinG, and RecU) (Fig. 2).

**Figure 4.**
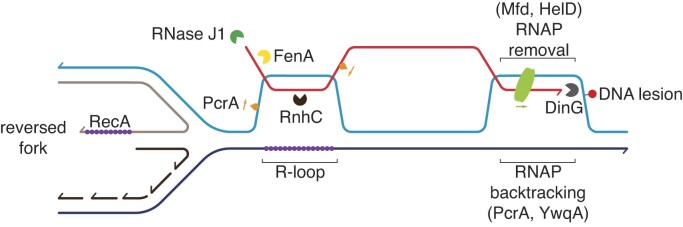
Cartoon showing how *B. subtilis* proteins may contribute to resolve RTCs. Here, a CD RTC is illustrated. A replisome clashes with multiple RNAPs transcribing highly expressed genes (i.e. rRNA genes), or the RNAP finds a DNA lesion on the template strand (red circle), and transcription is halted. Upon that, the stalled fork has a tendency to reverse and RNAP to backtrack, and this causes topological constrains that facilitate R-loop formation. RecA (purple circles) may bind to the ssDNA region in the regressed fork or in the R-loop. RNase J1 (green Pac-Man) or FenA (yellow Pac-Man) degrades the mRNA. RNAP and RecA, acting as hubs, interact with and recruit PcrA (orange drop), RnhC (black Pac-Man), or DinG (grey Pac-Man) at the trafficking conflict. PcrA displaces and RnhC degrades the RNA strand of the R-loop. PcrA or YwqA facilitates RNAP backtracking, Mfd or HelD facilitates RNAP removal and DinG degrades the exposed 3′-end of the mRNA to facilitate transcription reinitiation upon removal of the lesions. RecA bound to the reversed fork may protect it from degradation. Although not depicted here, topoisomerases may also play a role at RTC sites.

Single-cell analyses revealed that the replisome simply slowdown and skip CD transcription (Huang et al. 2023), but in highly expressed genes (as *rrn* loci) spontaneous replisome disassembly was observed in 40%–45% of total wt cells grown in rich medium (Mangiameli et al. 2017). RecA–GFP forms spontaneous foci in 15%–20% of total unstressed wt cells, with >85% of them colocalizing with DnaX (Simmons et al. 2007). DnaB, DnaD, and DnaC accumulate at the *rrn* operons in a RecA-dependent manner (Merrikh et al. 2011, Million-Weaver et al. 2015). Thus, RecA is also likely to accumulate at CD *rrn* loci where intrinsic RTCs occur. Once RecA is assembled at the site of RTC, it could stabilize PcrA, RnhC, or DinG by a direct protein–protein interaction. PcrA interacts with, and limits RecA filament growth, a process counterbalanced by RecO and SsbA (Fig. 4) (Sanders et al. 2017, Carrasco et al. 2022). *In vitro*, PcrA unwinds RNA–DNA hybrids (Moreno-Del Alamo et al. 2021, Urrutia-Irazabal et al. 2021).

The genetic analyses conducted thus far have revealed connections between these proteins and their putative involvement in resolving RTCs: (i) PcrA depletion inviability is suppressed by *recO* or *recA* inactivation (Moreno-Del Alamo et al. 2020, 2021); (ii) PcrA depletion lethality is exacerbated by *recU, recX, recD2, ywqA, helD, rnhC, dinG, recD2*, or *ywqA* inactivation, suggesting that these proteins could also act independently of PcrA to resolve RTCs (Moreno-Del Alamo et al. 2020, 2021); and (iii) *recA* or *recO* inactivation is synthetically lethal in the Δ*rnhC*, but not in the Δ*din*G context (Moreno-Del Alamo et al. 2020, 2021).

From biochemical data, we hypothesize how the mentioned translocases can contribute to *B. subtilis* RNAP recycling (Table 1 and Fig. 4). PcrA is a DNA translocase that binds to the RNA moiety and moving in the 3′→5′ direction disassembles R-loops (Carrasco et al. 2022). PcrA also interacts with and displaces RecA from ssDNA, and interacts with and backtracks and dislodges RNAP (Fig. 4) (Sanders et al. 2017, Carrasco et al. 2022). In fact, PcrA over-expression reduces RNAP ChIP signals at *rrn* operons (Yeesin 2019), suggesting a role for PcrA in dismantling RNAP from the DNA template at CD RTCs. Mfd binds to paused RNAP during transcription of structured RNA or at a DNA damage site, antibacktracks and physically removes stalled RNAP from the DNA (Ayora et al. 1996, Le et al. 2018, Ghodke et al. 2020, Ho et al. 2020, Ragheb et al. 2021). YwqA is believed to rescue a RTC by promoting RNAP backtracking as its *E. coli* homolog RapA does (Liu et al. 2015). HelD, in concert with the $\delta$ subunit of RNAP, displaces nucleic acids and contributes to RNA recycling, with ATP hydrolysis facilitating HelD detachment from RNAP (Fig. 4) (Wiedermannova et al. 2014, Newing et al. 2020, Pei et al. 2020). RecG may not unwind R-loops (Wen et al. 2005), and RecD2 and PriA have not been tested.

Among the transcription factors that physically interact with RNAP, NusA is essential for growth. NusA depletion affects the expression of *polC, dnaB, dnaD, dnaI, priA, recG, radA*, and *disA*, among others (Mondal et al. 2016). NusG, Rho and GreA are dispensable for growth. They may prevent misregulation of the replication stress response, but seem to have a minimal effect on RTCs (Johnson et al. 2020, Yakhnin et al. 2020, Wang and Artsimovitch 2021).

Proteins with nuclease activity may also contribute to cope with RTCs (Fig. 4). For instance, RnhC, interacts with and travels with RNAP even in the absence of exogenous DNA damage (Delumeau et al. 2011). RnhC senses, recognizes and removes the RNA portions of R-loops formed during RTCs, as well as RNA primers during Okazaki fragments maturation (Ohtani et al. 1999, Lang et al. 2017). DinG is a 3′→5′ exo(ribo)nuclease that may also participate in the resolution of RTCs (McRobbie et al. 2012, Carrasco et al. 2023). DinG may degrade the exposed 3′-end RNA upon RNAP backtracking, resulting in a 5′-ssDNA tailed RNA–DNA hybrid to which RnhC binds and removes the RNA (Fig. 4) (Carrasco et al. 2023). In fact, Δ*rnhC* or Δ*dinG* mutants further increase the lethality in PcrA-depleted cells (Moreno-Del Alamo et al. 2021). The FenA nuclease (also known as ExoR or YpcP) and Pol I 5′→3′ nucleases are recruited to replication forks upon DNA damage induction by UV light (Hernández-Tamayo et al. 2019). *In vitro*, FenA, and to a lesser extent Pol I, degrade several RNA:DNA hybrids, and participate in the removal of the RNA strand of persistent R-loops (Lowder and Simmons 2023). Another nuclease, RNase J1, predominantly colocalizes with RNAP. It functions as an endo- and 5′→3′ exoribonuclease, degrading the nascent RNA and disassembling the stalled RNAP upon collision through a “torpedo” mechanism (Sikova et al. 2020). RecA could mitigate RTCs by protecting the reversed fork and modulating the activities of nucleases and DNA helicases, although this remains to be analyzed (Fig. 4). Moreover, RecA activity at the site of RTC could be necessary for replication restart, because it is necessary for DnaD association, and thereby for DnaC loading, at RTCs (Million-Weaver et al. 2015).

Live cell studies have been also used to analyze HO RTCs. When an IPTG inducible *lacZ* gene was integrated in a HO orientation and expressed from a strong promoter, induction of transcription destabilized the replisome, and the number of cells containing two intact DnaC complexes significantly dropped, with >80% of cells containing only a single hexameric DnaC helicase in the replication factory (Mangiameli et al. 2017). Monoclonal S9.6-gold-based immune electron microscopy approaches have revealed the accumulation of RNA–DNA hybrids on HO conflicts in the Δ*rnhC* context (Stoy et al. 2023). With this engineered HO conflict, the stalled forks had a tendency to reverse (Stoy et al. 2023). Unwinding of DNA during transcription elongation generates positively supercoiled ahead and negatively supercoiled DNA behind RNAP (Liu and Wang 1987, Wu et al. 1988). The contribution of topisomerases to the resolution of RTCs remains poorly explored (Lang and Merrikh 2021). DNA topoisomerase I (Topo I or TopA) interacts with RNAP (Delumeau et al. 2011). Topo I depletion rescues *rrnIHG*-inverted cells from a severe growth defect when grown in LB medium (Yeesin 2019), although the molecular mechanism remains elusive. Topo II (*a.k.a*. DNA gyrase) and Topo IV, which are composed of the GyrA and GyrB, and the ParE and ParC subunits, respectively, were shown to preferentially associate to HO but not to CD RTCs, and are required for the removal of positive supercoils built up at HO RTCs (Lang and Merrikh 2021). Furthermore, DNA gyrase was found to drive pervasive R-loop formation, that should require replication restart (Lang and Merrikh 2021). Finally, whether Topo III is required to resolve RTCs in *B. subtilis* remains to be tested.

Live cell studies have revealed that RecA–GFP forms foci in ∼95% of *rrnIHG-*inverted cells, and these foci are RecO-dependent when grown in rich LB medium (Srivatsan et al. 2010). Inactivation of *recO, recR, recA, ruvB, recU*, or *rnhC* reduces viability (>2000-fold), but *recF* inactivation slightly decreases the plating efficiency of this strain when compared to the wt in LB medium (Yeesin 2019). RecA, which physically interacts with PcrA and RnhC (Carrasco et al. 2022, 2023) is required to ameliorate HO RTCs. Indeed, PcrA over-expression increases plating efficiency of *rrnIHG-*inverted cells grown in LB, but such effect is not observed in the absence of RecA. Contrarely, RnhC over-expression does not significantly affect the plating efficiency, both in the presence or the absence of RecA (Yeesin 2019).

Alleviation of RTCs in *E. coli* has been shown to occur differently. When the replisome collides with RNAP*_Eco_* on the template leading-strand, both machineries transiently pause. The Rep*_Eco_* and UvrD*_Eco_* translocases are recruited to the stalled fork, with Rep*_Eco_* interacting with DnaB*_Eco_*, and UvrD*_Eco_* interacting with an undefined partner in the replisome (Atkinson et al. 2011, Wollman et al. 2023). Then, Rep*_Eco_*, in concert with UvrD*_Eco_* or DinG*_Eco_*, primarily promotes the movement of replisomes through transcription complexes at CD RTCs (Guy et al. 2009, Boubakri et al. 2010, Hawkins et al. 2019, Whinn et al. 2023). When the *E. coli* replisome encounters a cluster of RNAPs on the template lagging-strand due to the inversion of the *rrnA* or the *rrnE* and *rrnB* operons, both machineries stall, leading to a HO RTC that does not significantly affect colony formation in wt, Δ*recA* or Δ*recG* cells plated on LB agar plates (Boubakri et al. 2010, De Septenville et al. 2012). In this scenario, there is pervasive disassembly of replisomes, that tend to reverse (Boubakri et al. 2010, De Septenville et al. 2012). The specific enzyme(s) that reverses the stalled forks remains unidentified (De Septenville et al. 2012). Remarkably, the resolution of RTCs at HO sites requires RecBCD*_Eco_*, or DinG*_Eco_*, but not RecA*_Eco_* (Boubakri et al. 2010, De Septenville et al. 2012).

## Future perspectives

Replication stress, which is an important source of genome instability, is an inherent challenge that DNA replication processes face due to the various obstacles encountered by the replisome, including stalled transcription machineries, bound proteins, and endogenous DNA damage. *B. subtilis* utilizes multiple mechanisms to coordinate rescue of stalled replication forks, and failure in this repair results in defects in transcription elongation, DNA damage and chromosomal segregation (Anderson et al. 2022). Cells have evolved a repertoire of strategies to handle replication stress, and the choice among these mechanisms can vary depending on the specific context and possible outcomes. Therefore, the multiple forms of differentiation and development of the *B. subtilis* bacterium may help us to define the proteins involved in different situations. Genetic works carried out over 50 years have contributed to our comprehension of the functions that contribute to cope with replication stress. Many proteins still remain to be analyzed, and our understanding of the DNA damage-dependent but SOS-independent regulation is yet poor, due to the intricate interplay of multiple regulators.

In the past two decades, single-molecule fluorescence observations, genomic and proteomic analyses in live bacteria, and biochemical studies have helped us to the reconstruction of mechanisms employed by cells to manage replication stress and RTCs. These investigations are unraveling the pathways chosen by cells, and the roles of the various proteins involved in these intricate processes. It is conceivable that future studies will benefit from methodologies aimed at determining helicase loading and dynamics in response to diverse types of replication stress, and the contribution of error-prone TLS polymerases, which is still poorly understood in *B. subtilis* cells.

Another critical area of future investigation is the comprehension of the biological contribution of noncanonical RecA activities to the choice of the pathway to alleviate replication stress, and the coordination of the checkpoints and fork remodelers. The identification and study of mutants in which different RecA activities are specifically inactivated should help us to understand noncanonical RecA mechanisms. Numerous protein–protein interactions among the different functions that mitigate replication stress have been documented, but structural information of assembled complexes is still needed to fully understand these interactions and how they are coordinated. It is also necessary to unravel the contribution in response to replication stress of secondary metabolites ([p]ppGpp, c-di-AMP, and potentially unidentified compounds, such as the one synthesized by CczA; Wozniak et al. 2022), as well as the effect of levels of replication proteins and dNTPs imbalance. Furthermore, understanding the loading of primosomal proteins (DnaC, DnaG, and DnaE) during replication restart, and their particular coordination on hybrid primer synthesis on the nascent lagging-strand, requires further studies.

*Bacillus subtilis* serves as a valuable model for deciphering the molecular mechanisms and the crucial proteins involved in overcoming replication stress *via* error-free DDT in pathogenic bacteria of the *Bacilli* Class (*Staphylococcus, Streptococcus, Enterococcus*, and so on). A better overview of these processes could help us to develop novel targets for the development of safe and effective antimicrobial agents.

## Footnotes

1 Unless stated otherwise, the indicated genes and products are of *B. subtilis* origin. The nomenclature used to denote the origin of proteins from other bacterium is based on its Genus and species (e.g. *Escherichia coli* DnaB is referred as DnaB*_Eco_*).
